# Proof of concept of faecal egg nematode counting as a practical means of veterinary engagement with planned livestock health management in a lower income country

**DOI:** 10.1186/s13620-017-0094-9

**Published:** 2017-06-02

**Authors:** Eithne Leahy, Barend Bronsvoort, Luke Gamble, Andrew Gibson, Henderson Kaponda, Dagmar Mayer, Stella Mazeri, Kate Shervell, Neil Sargison

**Affiliations:** 1 Worldwide Veterinary Service, 14 Wimborne Street, Cranborne, Dorset BH21 5PP UK; 2 Royal (Dick) School of Veterinary Studies, Easter Bush Veterinary Centre, University of Edinburgh, Roslin, Midlothian EH25 9RG UK; 3 The Epidemiology, Economics and Risk Assessment (EERA) Group, The Roslin Institute, Easter Bush, Roslin, Midlothian EH26 9RG UK; 4 Mission Rabies, Cranborne, Dorset UK; 5 Blantyre District Agricultural Development Office, Kunthembwe EPA, Malawi

## Abstract

**Background:**

The wellbeing and livelihood of farmers in impoverished regions of the world is intrinsically linked to the health and welfare of their livestock; hence improved animal health is a pragmatic component of poverty alleviation. Prerequisite knowledge and understanding of the animal health challenges facing cattle keepers in Malawi is constrained by the lack of veterinary infrastructure, which inevitably accompanies under-resourced rural development in a poor country.

**Methods:**

We collaborated with public and private paraveterinary services to locate 62 village Zebu calves and 60 dairy co-operative calves dispersed over a wide geographical area. All calves were visited twice about 2 to 3 weeks apart, when they were clinically examined and faecal samples were collected. The calves were treated with 7.5 mg/kg of a locally-available albendazole drench on the first visit, and pre- and post- treatment trichostrongyle and *Toxocara* faecal egg counts were performed using a modified McMaster method.

**Results:**

Our clinical findings point towards a generally poor level of animal health, implying a role of ticks and tick-transmitted diseases in village calves and need for improvement in neonatal calf husbandry in the dairy co-operative holdings. High faecal trichostrongyle egg counts were not intuitive, based on our interpretation of the animal management information that was provided. This shows the need for better understanding of nematode parasite epidemiology within the context of local husbandry and environmental conditions. The albendazole anthelmintic was effective against *Toxocara*, while efficacy against trichostrongyle nematodes was poor in both village and dairy co-operative calves, demonstrating the need for further research to inform sustainable drug use.

**Conclusions:**

Here we describe the potential value of faecal nematode egg counting as a platform for communicating with and gaining access to cattle keepers and their animals, respectively, in southern Malawi, with the aim of providing informative background knowledge and understanding that may aid in the establishment of effective veterinary services in an under-resourced community.

## Background

In setting out its agenda for sustainable development, the Food and Agriculture Organisation of the United Nations has established specific goals, offering a vision of a fairer, more prosperous, peaceful and sustainable world in which no one is left behind [1]. These goals emphasise the need for improvement in the socioeconomic sustainability and efficiency of integrated livestock agriculture in order to meet the food requirements of the world’s population. However, the goal of livestock agriculture in alleviating poverty in poor African countries is seldom achieved, despite independent global advances in animal breeding and genetics, sustainable husbandry and health management [2]. The failure to translate these advances into efficient utilisation of natural resources by livestock in local target environments must be understood before it can be addressed through knowledge transfer and applied research. Furthermore, impoverished people who depend upon their animals for subsistence and wellbeing face zoonotic disease risks. Hence, better animal health and welfare aids human wellbeing by addressing food security and ensuring a healthy diet, having socio-economic and environmental benefits, and improving health standards through the control of zoonoses [3].

Poverty and malnutrition in southern Africa is most apparent in rural regions where people depend upon agriculture for subsistence. Malawi is one of the poorest countries in southern Africa with amongst the lowest gross national incomes per capita (ppp international $, 2013: 750) and human life expectancy at birth (male: 57 years, female: 60 years) in the world [4]. More than half of the population lives below the poverty line, and over 85% depend on subsistence agriculture. Poor rural people in Malawi are unable to diversify out of agriculture and tend to remain underemployed for part of the year due to the seasonal nature of agricultural production. Village livestock production is predominantly derived from poultry, meat-producing goats, indicine cattle, and pigs. Livestock husbandry and crop production are integrated, with devolved responsibility of individuals for the care and management of different groups of animals. Livestock performance is affected by low productivity of the cropping sector; and as cropping extends into grazing areas, numbers of ruminant livestock have been decreasing [5]. Livestock in southern Malawi are communally managed, but generally individually owned by community members. An obvious route towards poverty alleviation and improved human health, therefore, lies in improved livestock production efficiency and organisation and equitable markets [6].

About 80% of cattle production in Malawi is dependent on extensive communal grazing, in which Zebu cattle roam freely during the day and are housed at night in pens (referred to as kholas) constructed in the villages. Ownership of cattle is well defined, and in the absence of contemporary financial systems, or marketing infrastructures, they are valued in terms of economic unit numbers ahead of being income-generating resources. Cattle belonging to different individuals are co-grazed and taken to water on grounds approved by a headman acting under the authority of the village chief. Arable cultivation rights are granted in a similar manner. Village cattle are slaughtered at varying ages for different reasons. They are walked to the nearest slaughter slab and slaughtered by local butcher men using the Halal method.

In Malawi, the dairy value chain has been identified as a potential area to develop markets as a means of poverty reduction [7], targeting poor women through extension and development project support [8]. Co-operative dairy production from taurine cattle is operated under the auspices of regional dairy associations, which operate milk buying groups where landless cow keeper smallholders bring their milk daily to be cooled and collected for processing before sale to the urban public [9]. Typically producers are women who own a single cow, and can generate additional income through calf or heifer sales. The system has clear socioeconomic benefits by providing women with security through financial income generating responsibility.

The Malawian Government is responsible for notifiable and transboundary animal disease control [10]. Official veterinary services are regulated under the Department of Animal Health and Livestock Development (DAHLD) who are responsible for the training of Assistant Veterinary Officers (AVOs). AVOs are trained in basic animal health at a paraveterinary level, and are each responsible for providing services to farmers in one of about 150 Extension Planning Areas (EPAs). Transport links to remote villages are generally poor, becoming impossible during the rainy season, while use of medicines for the prevention of disease and treatment of sick animals is constrained by cost and availability. The poor veterinary infrastructure, combined with logistical challenges, deprive AVOs of the support that would enable them to be involved with planned livestock health management. As a consequence of the rudimentary nature of veterinary services, levels of livestock production are unknown, while the primary animal health constraints are not understood. A similar lack of baseline data on the dynamics and impact of livestock disease pertains to other parts of Africa [11].

Sub-tropical climates such as that in Malawi provide seasonal opportunities for arthropod, protozoa and helminth parasites, which have free-living environmental stages or vectors. The epidemiology, and hence appropriate management of these infections is influenced by climatic variation. Parasite infestations are seen at local slaughter slabs, hence livestock owners are generally aware of their presence and of associated costs and are receptive towards improved control regimes. Parasitic infestations are generally amenable to simple diagnostic procedures, hence engagement with parasite control, involving practical diagnostic tests, is a globally recognised means of engendering broader, holistic planned animal health management [12].

In this study, we adopted a pragmatic approach exploiting the investigation of nematode parasitology in calves to enable us to visit villagers and dairy co-operative cow keepers, to examine their calves, discuss their health management and understand the veterinary infrastructure in the context of the southern part of Malawi. The aim of our study was to show the value of practical parasitology in identifying opportunities to improve the efficiency of cattle production, with particular reference to those animal health problems for which there are known solutions. Our approach provided information that may help to inform the nature of relationships between nematode parasitism and co-infections with other diseases [13].

## Methods

### Resources

The study was undertaken between May and August 2016 in the rural area of the Blantyre District in southern Malawi. Working with local animal health networks afforded a unique insight to village and dairy co-operative farming systems, and provided interpretation of the local Chichewa language.

**Fig. 1:**
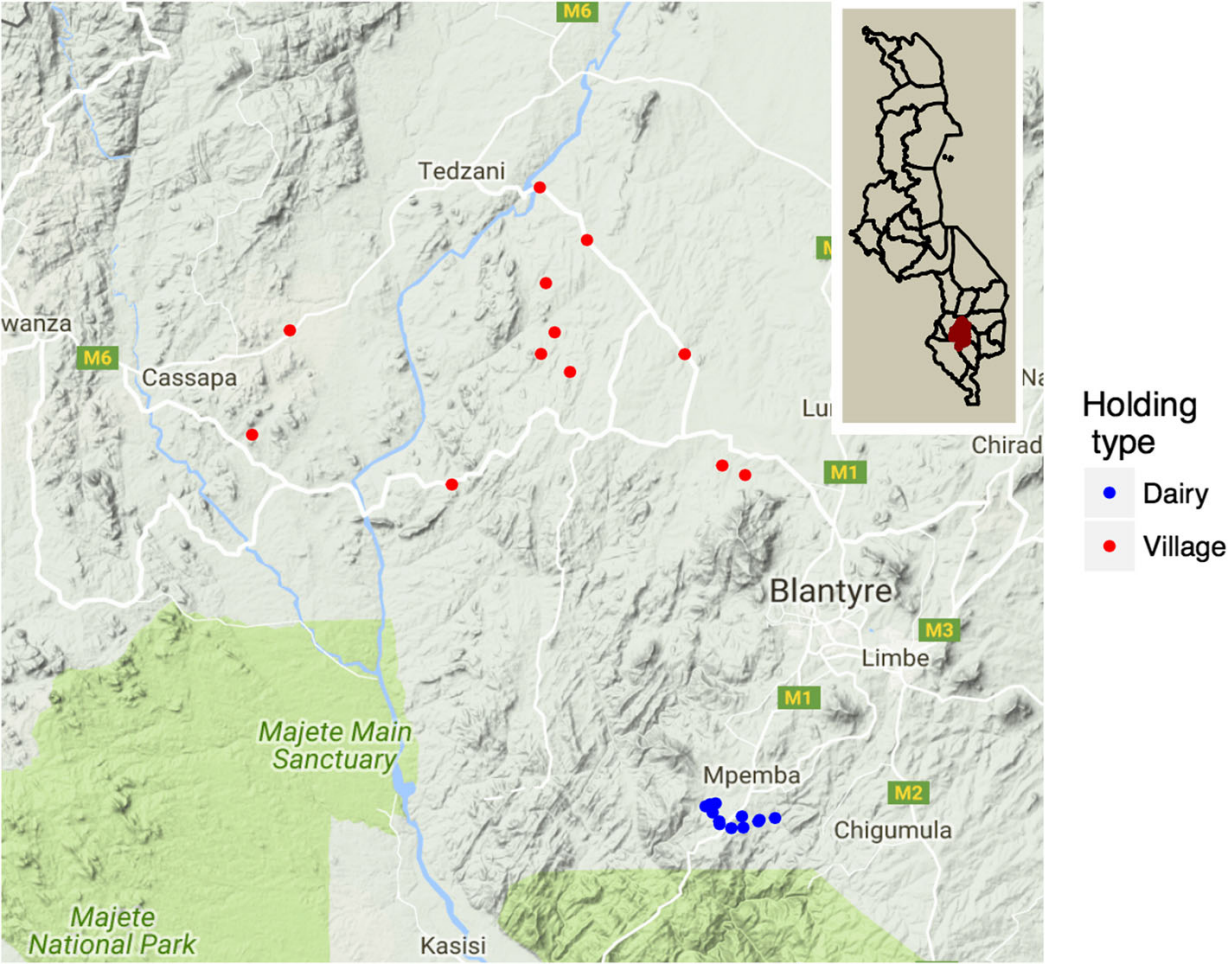
Map showing the locations of the villages and holdings where dairy calves were kept. Inset: outline map of Malawi. The study region is very small and not necessarily representative of the whole country. The map shows the geographical dispersal of the villages, while dairy co-operative holdings are clustered close to transport links to Blantyre city

Visits to 12 villages (Fig. 1) were arranged by the AVO and vet scouts with responsibility for the Kuthembwe area, which is the largest of the five EPAs within the Blantyre District. When health issues arise in village livestock, farmers or traditional village authorities usually contact a government-recognised person living within the community, known as a vet scout or Community Animal Health Worker (CAHW). These individuals report to the nearest AVO. Vet scouts or AVOs purchase limited supplies of animal remedies from wholesale shops when available and distribute them for varying fees to vet scouts or CAHWs who, in turn, administer medicines to animals. Communication between the AVOs, vet scouts, traditional village leaders and farmers was key in order to gain permission to access calves and also to physically locate calves in rural areas where herds are extensively grazed and dispersed.

Visits to dairy co-operative cattle were arranged through the Shire Hills Milk Producers Association (SHMPA), working within their Mpemba Bulk Tank Group situated within relatively easy access of Blantyre city (Fig. 1). SHMPA is the largest dairy co-operative in Malawi with over 6,500 milk producing smallholders. Cows are hand milked and raw milk is taken daily to bulk milk tanks, which normally fall within a 5 km distance from the holdings. Milk is collected every 48 h to be transported to a pasteurisation unit. SHMPA also oversees a high density stock farm from where heifers are sourced for individual farmers. Cows are always permanently confined in kholas and zero grazed on cut herbage (cut and carry) with supplementary feeding of brewery by-product concentrates. SHMPA operates an independent medicine supply and artificial insemination service which is undertaken by 33 Farm Livestock Technicians (FLTs). FLTs are the main providers of paraveterinary services. Communication between the FLTs and dairy co-operative smallholders was key in order to gain access to the calves.

### Study design

Calves from 1 to 6 months-old were recruited into the study from 15 smallholder dairy cattle locations (60 calves, mostly kept individually) and 12 villages (62 calves) (Table 1) during June and July 2016. Each calf was subjected to a standardised veterinary clinical examination [12], and all findings recorded on paper before transferring to a database (Microsoft Excel). The girth measurements of each calf were recorded using a locally-sourced weight band (unbranded), providing a crude estimate of the animals’ weights [14]. Villages and dairy co-operative smallholders were re-visited 13 to 24 days later and calves were re-examined.

**Table 1:** Number of cows of reproductive age and calves present in each village at the time of the first visit

Village	Cows	Calves
Andiseni	5	5
Chaswanthaka	11	7
Chinkango	7	4
Gwadani	20	10
Kaliwati	10	4
Kanyanda	5	4
M’banda	4	2
Maneya	9	4
Mkata	9	8
Mtenje	9	7
Mtiza	7	3
Ngoleka	13	4

Once calves were located, the farmer’s name, when known, and address were recorded and the coordinates of the village or khola location were geo-referenced. Each calf was photographed on both visits using a smart phone and given a unique identification number. A full clinical history was taken on both visits, recording any previous treatments or interventions. In the case of the village calves, it was also noted if any other animals within the herd had been unwell, or received any treatment. This information was based only on the memory of the livestock keeper, village leader, vet scout, or CAHW due to a lack of written records. Information on the health of dairy cattle and milk production levels was accessed from mostly written records.

### Parasitology

On both visits, faecal samples were collected into plastic bags from which air was excluded and then transported to Blantyre city for faecal egg counting on the same day. Samples were collected from 62 village and 60 dairy co-operative calves on the first visit and from 60 village and 56 dairy co-operative calves on the second visit, 13 to 24 days later. Faecal nematode egg counts (FECs) were determined using a saturated saline floatation modified McMaster method with a sensitivity of 50 eggs per gram (epg) [15].

Calves were treated with 7.5 mg/kg of a locally available albendazole (Alben Blue 10%; Vetagro, Kenya) at the time of the first visit. Reductions in faecal egg counts after treatment [16, 17] were calculated for 39 village and 39 dairy co-operative calves that were re-sampled 13 or 14 days after anthelmintic treatment. Post-treatment reductions in arithmetic mean FECs and 95% confidence intervals were calculated using the web interface package ‘*eggCounts’* [18] in R (Version 3.0.3) [19].

Ticks were identified by referring to standard keys, textbook descriptions and undergraduate teaching materials.

## Results

### Resources

Of the 62 village calves examined on the first visit, two were reported as missing at the time of the second visit, with a suggestion that they had been used as loans. For simplicity the missing animals were removed from the study analysis. None of the village calves had previously been treated with any animal remedy. Of the 12 villages included in the study only 2 reported any intervention from a vet scout to treat an animal in the herd since the previous rainy season (during which communication is almost impossible). No AVO had previously treated any species of animal in the 12 villages. (5 village calves were treated with amitraz between the first and second visits, as a consequence of our intervention.)

The number of cows of reproductive age in each of the villages that was visited is shown in Table 1. The average daily distance walked from khola to drinking water was 4.6 (range 2–6) km each way. The average measured distance by road from the sampled villages to a slaughter slab was 7.7 (range 3–15) km. There was no seasonal calving pattern, and little appreciation for the importance of reproductive management or production targets.

Of the 60 dairy calves examined on the first visit, one calf was reported as dead and three calves were reported as missing at the time of the second visit. For simplicity the dead and missing animals were removed from the study analysis. Twenty four dairy calves were reported to have received some form of previous treatment. Eighteen of these were located at the high density heifer rearing farm. Treatment records showed periodic use of albendazole, oxytetracycline, amitraz and deltamethrin, sourced from the dairy co-operative and administered by a farm worker, and a single ivermectin dose administered by a FLT. (17 dairy calves, all on the heifer rearing farm, were treated with amitraz and 12 were treated with cypermethrin between the first and second visits.) The concept of drug residues in food and withdrawal periods was not understood among cattle keepers.

**Fig. 2:**
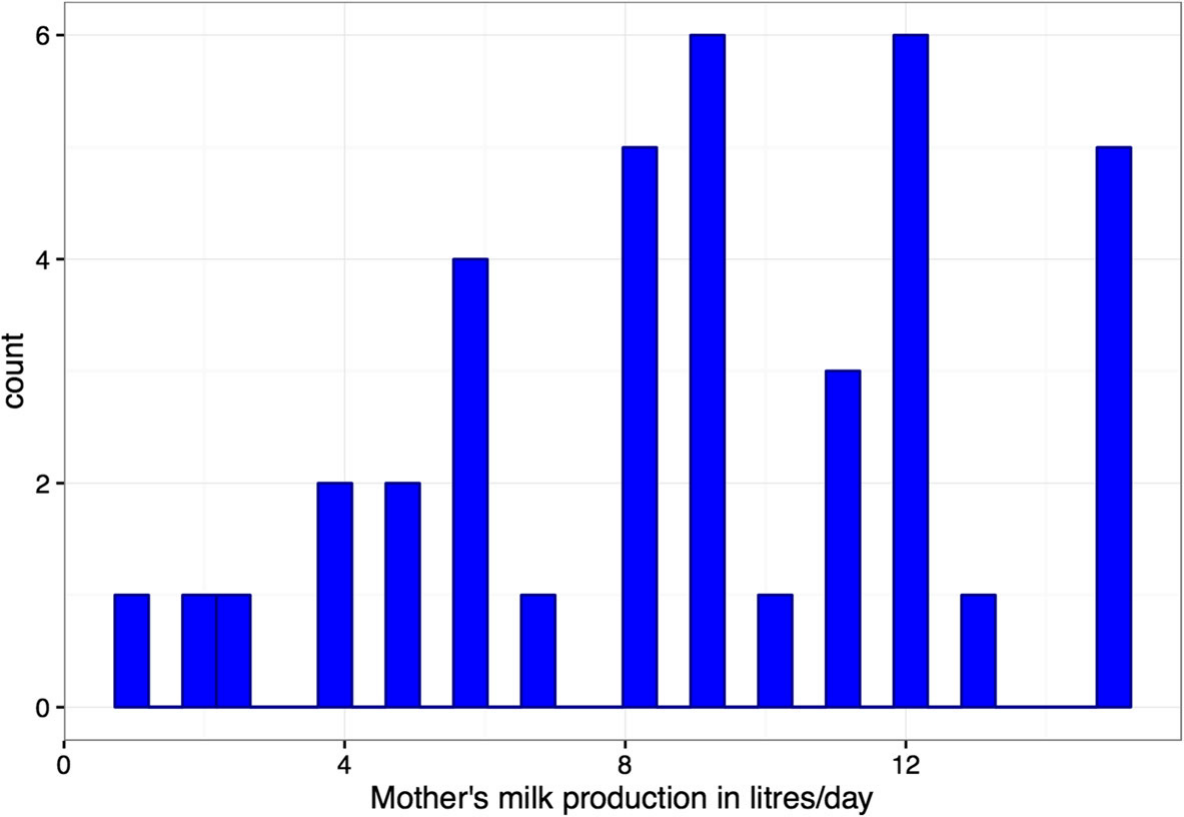
Daily milk yields of dairy co-operative cows on holdings where calves were visited for which data were available

The number of cows of reproductive age in each of the kholas and on the farm where dairy co-operative calves were visited is shown in Table 1. The average daily production of milk (litres per day) from 15 dairy cows (excluding 3 cows which were not lactating) present on the dairy co-operative holdings (Table 1) was 9.0 (range 1 to 15) (Fig. 2). Failure of cows to rebreed was perceived to be the major constraint to production.

### Clinical findings

**Fig. 3:**
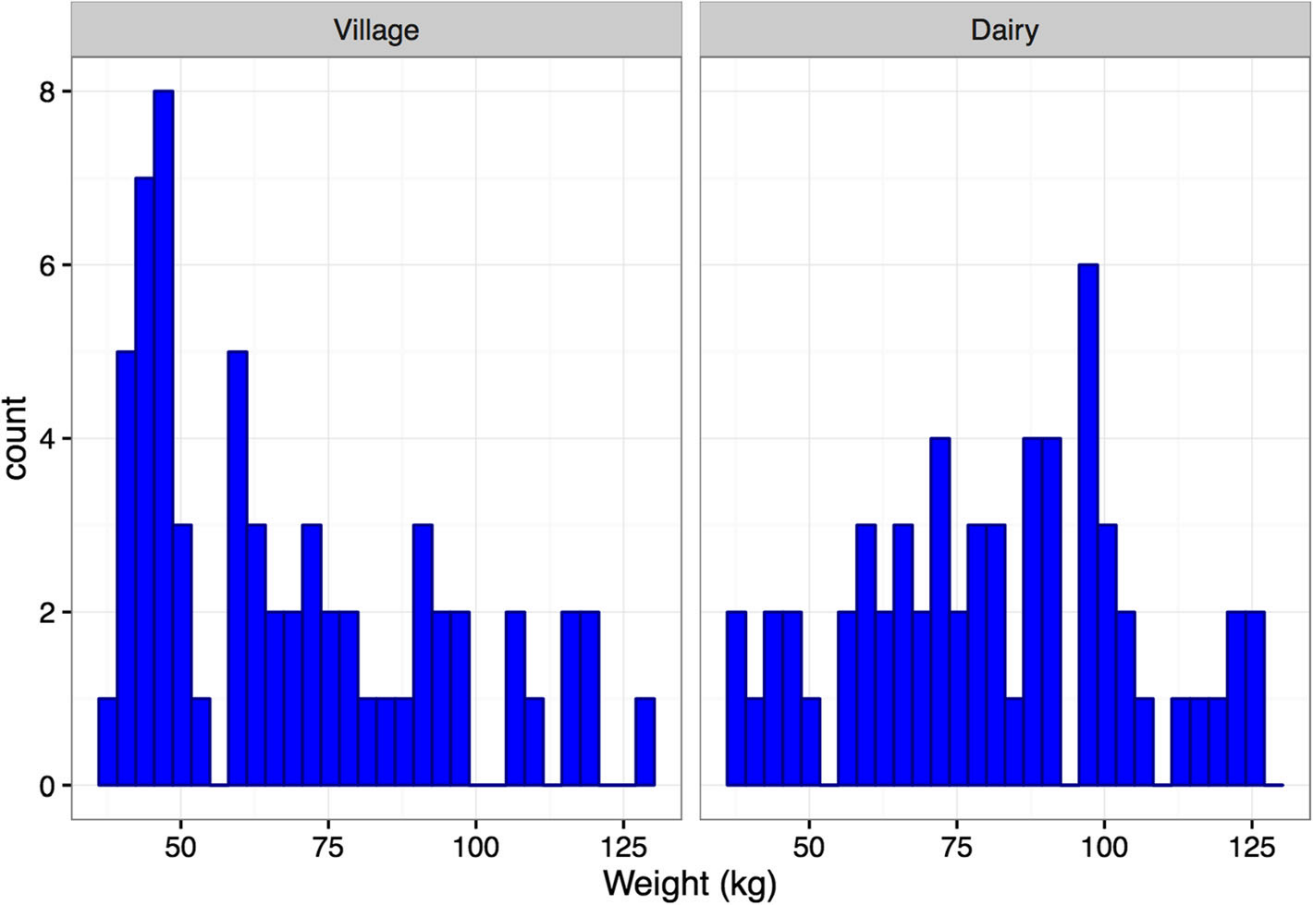
Estimated calf weights based on translation of girth measurement (cm) to kg using a locally-sourced weight band. The accuracy of this estimation is challenged, as the weights appear to exceed reasonable targets for cattle of corresponding ages

32 female and 30 male village calves, and 41 female and 19 male dairy co-operative calves were visited. Based on the owners’ recollection, the mean estimated age on the first visit of the 62 Zebu village calves was 3.61 months, and the mean age of 45 Friesian cross and 15 Jersey cross dairy calves was 3.92 months. The mean estimated weights of the village and dairy co-operative calves at the first visit were 68.7 kg and 81.1 kg, respectively (Fig. 3).

**Fig. 4:**
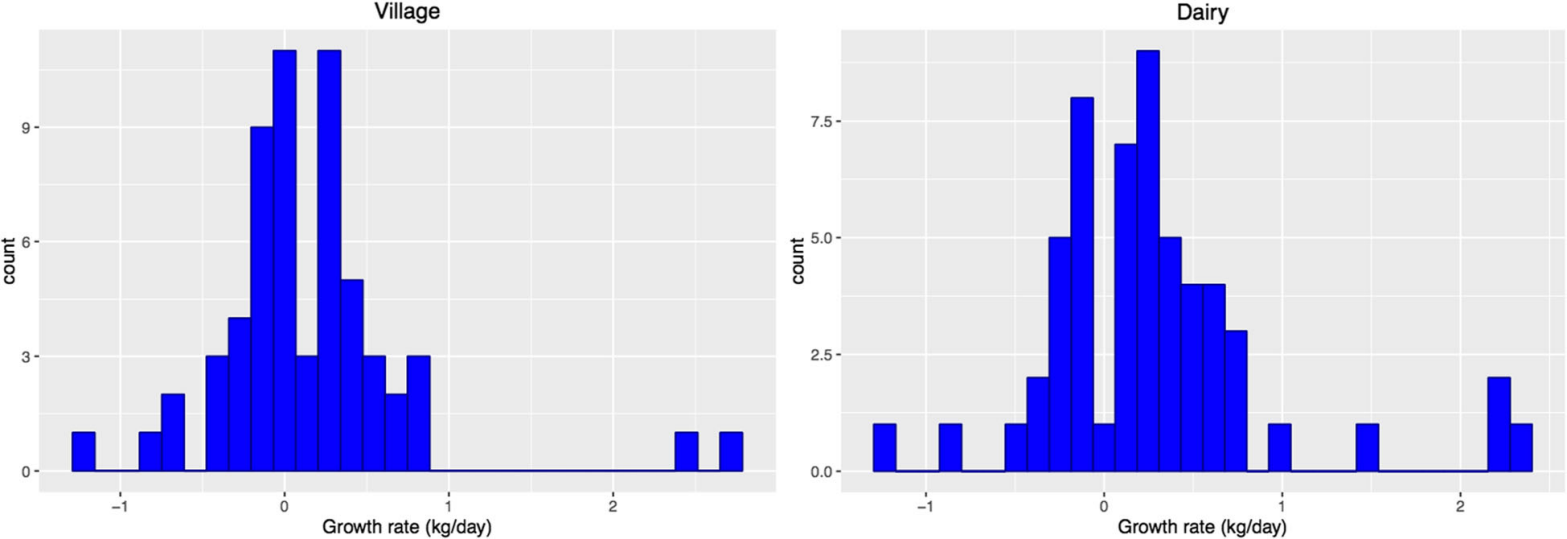
Daily live weight gains of dairy co-operative and village calves

The crudely estimated daily live weight gains of the village and dairy co-operative calves between the first and second visits were 0.16 kg/day and 0.28 kg/day, respectively (Fig. 4).

Pertinent findings identified on clinical examination of the village and dairy co-operative calves on each visit are summarised in Table 2. The most notable clinical findings in the village calves were a dull coat with multifocal alopecia, tick burdens, pale mucous membranes and enlarged peripheral lymph nodes. Ticks were morphologically identified as being *Rhipicephalus* spp. (found on the ears), *Hyalomma* spp. (found around the anus and feet) and *Rhipicephalus* (*Boophilus*) spp. (found mostly over the head and neck). Dull coats and alopecia were also present in the dairy co-operative calves, but the prevalence of tick burdens, pale mucous membranes and enlarged peripheral lymph nodes was lower than in the village calves. The proportions of calves with signs of diarrhoea, swollen joints and hard navels were higher in the dairy co-operative than in the village calves. Intriguingly, heart murmurs were identified in several dairy co-operative and village calves.

**Table 2:** Proportions of village and dairy co-operative calves showing pertinent clinical signs during each visit

	Visit 1		Visit 2	
	Village	Dairy co-operative	Village	Dairy co-operative
dull coat	0.53	0.75	0.70	0.83
presence of alopecia	0.73	0.77	0.63	0.66
evidence of diarrhoea	0.06	0.30	0.12	0.38
swollen joints	0.02	0.08	0.02	0.07
enlarged navel	0.16	0.20	0.2	0.29
enlarged peripheral lymph nodes	0.73	0.28	0.85	0.23
pale mucous membranes	0.66	0.30	0.8	0.34
ticks seen	0.61	0.08	0.4	0.05
nasal discharge	0.05	0.08	0.12	0.04
abnormal lung sounds	0	0.07	0.02	0.02
heart murmur on auscultation	0.26	0.20	0.47	0.36
conjunctivitis	0.03	0.07	0.06	0.08
presence of mouth ulcers	0.05	0	0.05	0
traumatic eye lesions	0.05	0.02	0.03	0.02
dehydration	0.21	0.10	0.24	0.15

### Summary parasitology data for all of the calves (shown without reference to anthelmintic efficacy)

The trichostrongyle and *Toxocara* FECs of the village and dairy co-operative calves included in the study, before and 13 to 24 days after oral treatment with 7.5 mg/kg albendazole are shown in Table 3. The results show high pre-treatment trichostrongyle and *Toxocara* FECs in village calves, and lower but nonetheless positive trichostrongyle and *Toxocara* FECs in dairy co-operative calves. However, 17/60 and 26/56 of the village and dairy calves, respectively, had pre-treatment trichostrongyle FECs of zero. 33/60 and 51/56 of the village and dairy calves, respectively, had pre-treatment *Toxocara* FECs of zero.

**Table 3:** Summary of trichostrongyle and Toxocara FECs of all of the village and dairy co-operative calves

Group	Number of calves	Mean (±SEM) and median (range) trichostrongyle epg before treatment	Mean (±SEM) and median (range) trichostrongyle epg after treatment	Mean (±SEM) and median (range) *Toxocara* epg before treatment	Mean (±SEM) and median (range) *Toxocara* epg after treatment
Village	30 female 30 male	652 (±233) 150 (0 – 11,950)	88 (±19) 50 (0 – 900)	2,910 (±1,168) 0 (0 – 50,600)	4 (±2) 0 (0 – 100)
Dairy co-operative	38 female 18 male	93 (±26) 50 (0 – 1,350)	79 (±22) 25 (0 – 1,050)	209 (±197) 0 (0 – 11,050)	4 (±3) 0 (0 – 100)

These data were not used to show drug efficacy, as the second samples were collected from some of the calves after a time interval that exceeded the minimum prepatent period of 17 days for most parasitic trichostrongyle nematodes. Nevertheless data can be used to confirm benzimidazole treatment efficacy against *Toxocara* in infected calves in both groups.

### Parasitology data for calves that were sampled to evaluate anthelmintic efficacy

The mean (±SEM) pre and post-treatment FECs of only the dairy co-operative and village calves that were revisited 13 or 14 days after oral treatment with 7.5 mg/kg albendazole, to allow the efficacy of the drug to be evaluated are shown in Table 4. The results show poor efficacy of the albendazole anthelmintic against trichostrongyle nematodes in both groups of calves. The results confirm efficacy of the benzimidazole drug against *Toxocara* in the village calves, but are less informative for the dairy calves due to the small numbers having measurable pre-treatment *Toxocara* FECs.

**Table 4:** Mean (±SEM) FECs (epg) of the 1 to 6-month old village and dairy co-operative calves (mean age at first visit: 3.91 and 3.95 months, respectively) that were sampled before and 13 or 14 days after oral treatment with 7.5 mg/kg albendazole to evaluate anthelmintic efficacy

Group	Number of calves	Pre-treatment trichostrongyle epg	Post-treatment trichostrongyle epg	Pre-treatment *Toxocara* epg	Post-treatment *Toxocara* epg
Village	21 female 18 male	778 (±335)	81 (±17)	1559 (±1027)	4 (±3)
		FECR 95% CIs:	89.6% 72.6%; 96.1%	FECR 95% CIs:	99.8% 98.2%; 100%
Dairy co-operative	26 female 14 male	106 (±34)	105 (±30)	286 (±276)	4 (±3)
		FECR 95% CIs:	1.2% −132.1%; 57.9%	FECR 95% CIs:	98.7% 85.3%; 99.9%

**Fig. 5:**
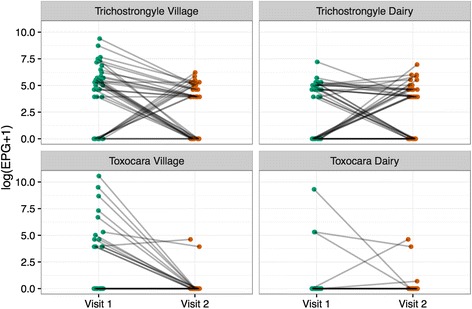
Log FEC + 1 of 40 individual dairy co-operative and 39 individual village calves before and 13 or 14 days after oral treatment with 7.5 mg/kg albendazole

The two calf populations used for the analysis of anthelmintic efficacy are derived from 7 villages and 13 smallholder dairy cattle locations, and the distribution of egg counts is both aggregated and overdispersed within each calf population. Furthermore, some animals had pre treatment FECs of 0 epg and positive post-treatment FECs. Pre- and post-treatment FECs of individual village and dairy co-operative calves are represented in Fig. 5 to illustrate these points, and to graphically depict patterns for changes in pre- and post-treatment trichostrongyle egg counts.

## Discussion

Malawi lacks core veterinary infrastructure. There is no Government system of veterinary practice in the country, and no existing active disease surveillance. To-date, there have not been any health surveys of the country’s ruminant livestock, or objective evaluation of the efficiency and sustainability of current systems, as have been undertaken in other low income African countries [20, 21]. These are fundamental requirements to improve the efficiency of Malawian livestock agriculture and ensure sustainable food production. A lack of future planned animal health management will impede progress and hold back successful farmers who have the potential and vision to succeed.

Our small-scale study shows the value of using nematode parasitology as a means of engaging with livestock keepers in a small region relatively close to Blantyre city, allowing a unique preliminary insight to the health status of Malawi’s cattle. Evaluation of tick burdens might have afforded an alternative means of engagement, albeit subsequent interventions would have been challenging due to community dip tanks having fallen into states of disrepair. The action of locating, catching, restraining and clinically examining each calf that was recruited to the parasitological study maximised time spent with their keepers, providing a chance to exchange knowledge in an enthusiastic and open-minded manner. A larger study involving many more village and dairy co-operative calves distributed throughout the entire country would have been needed to provide irrefutable information about animal health priorities, but was not possible due to the lack of animal health organisation and logistical challenges in Malawi, including the wide geographical dispersal and remoteness of small groups of cattle, poor rural administrative infrastructures, and poor transport and communication networks. Furthermore, direct collection of production data would have been potentially awkward due to the sensitivities surrounding livestock being personal investments ahead of food producing resources.

A wider survey of nematode parasitism and evaluation the efficacy of anthelmintic drug treatment is needed to gather and analyse epidemiological data that is necessary to place our parasitological results into the context of animal husbandry, environmental conditions, animal health and potential co-infections. The low numbers of calves relative to the geographical area used in our study, the small numbers of adult cattle per village, and the large distances that village cattle must walk daily to water, are indicative of a low output system based on extensive grazing on natural herbage. Conversely, individual co-operative dairy cattle are more intensively managed and kept within easy reach of milk collection centres that are situated within accessible transport links to Blantyre city.

The village cattle keepers informed us that they had very limited access to conventional veterinary medicines, which were considered to be unaffordable. Albendazole given as part of our study, and amitraz that was administered to five calves following our identification of massive tick burdens, were reported to have been the only treatments ever administered to Zebu calves in the villages that we visited. Only two of the twelve villages that we visited reported any form of paraveterinary contact, highlighting the almost total absence of informed animal health care or disease mitigation. Access to conventional veterinary medicines and paraveterinary advice was available to the smallholder dairy co-operative cattle keepers. However, this situation may have given rise to the *ad hoc* and potentially unsustainable use of remedies.

The attrition rate of calves from the study between the first and second visit, having died or been used as loans, coupled to poor growth rates and the unhealthy appearance of the animals on clinical examination is concerning. The calf weights extrapolated from the weight band were intuitively inaccurate, while the estimates of growth rates comparing measurements taken with the same weight band are more likely to be informative. The clinical findings in both the village and dairy co-operative calves are suggestive of these individual animals being co-infected with multiple pathogens, as shown in previous African studies [13].

The predominant problem in the dairy calves of diarrhoea, and identification of animals with swollen joints and navels subjectively suggest poor passive colostral transfer of immunity and poor hygiene, although this differs from the objective findings of a previous study of passive antibody transfer in calves in western Kenya [22]. Among the dairy co-operative smallholders in our study, little importance was given to colostrum, which many perceived as being ‘sour milk’ owing to its failing alcohol quality control tests and being rejected from bulk milk tanks.

The predominant signs seen in the village calves of pale mucous membranes, peripheral lymphadenopathy and the high prevalence of tick infestation highlight the importance of tick transmitted diseases. This situation is known to be commonplace throughout southern and eastern Africa [23]. Neonatal calf husbandry and tick control would, therefore, be pragmatic starting points for planned animal health management of the dairy co-operative and village cattle, respectively.

High pre-treatment *Toxoacara* FECs (greater than an arbitrary value of 1,000 epg) in eleven of the village calves, but only one dairy co-operative calf may reflect different levels of exposure of their free-ranging and confined dams to infective eggs. The overall production limiting impact of adult *Toxocara vitulorum* infection in young calves is poorly defined. Heavy burdens giving rise to high FECs cause ill thrift, dry coat appearance and intermittent diarrhea, with occasional deaths resulting from intestinal obstruction and peritonitis [24, 25]. The zoonotic potential of *T. vitulorum* to cause of human visceral larval migrans in a comparable manner to *Toxocara canis* [26] and *Toxocara cati* [27] is not known [28, 29], but nonetheless noteworthy [30]. In our study, the locally available albendazole drench at a dose rate of 7.5 mg/kg was effective against *T. vitulorum*, demonstrating a potential opportunity for improved health management of village calves [31], for example involving benzimidazole treatments at 10 to 14 days after birth.

The presence of trichostrongyle eggs in the faeces of 30 of the 60 dairy calves was unexpected, as they were reportedly generally confined to the kholas. Extrapolating from knowledge of managed production systems using taurine cattle [32], the trichostrongyle FECs of the village calves were also higher than expected for young suckled calves grazing alongside immune adult animals. Median FECs of 200 epg (range 0 – 12,250 epg), compared with 150 epg (range 0 – 11,950 epg) in our study were reported in similar aged Zebu calves in western Kenya [33]. Furthermore, our samples were collected in the dry season, when survival of infective larvae on herbage may have been short [32], while the extensive grazing management would result in low levels of challenge. There may, therefore, be areas that are permanently heavily contaminated with infective larvae on wetter pastures close the water sources, where different groups of cattle come together, although this is not known. The identification of counter-intuitive trichostrongyle nematode burdens raises opportunities for further studies and iterative animal health planning.

Taken at face value, the percentage reductions in trichostrongyle FECs before and 13 to 14 days after treatment with 7.5 mg/kg albendazole suggest notably poorer anthelmintic efficacy against trichostrongyle nematodes in the dairy co-operative calves (1.2% FECR) than in the village calves (89.6% FECR). However, the 95% confidence intervals calculated using the ‘eggCounts’ web interface [18] for the percentage reductions in trichostrongyle FECs do not provide meaningful statistical support for comparisons in anthelmintic efficacy between the two populations, each made up of several smaller groups in which FECs were aggregated and overdispersed [34]. The graphic depictions of raw pre- and post-treatment FEC data (Fig. 5) illustrate the manner in which differences in the level of efficacy of the albendazole anthelmintic between the two populations (Table 4) are influenced by data from individual animals. The two calf populations used for the analysis of anthelmintic efficacy were derived from 7 villages and 13 smallholder dairy cattle locations, each with potentially different animal, environmental and management factors influencing parasite challenge and burdens. The modified McMaster faecal egg counting method used (MAFF, 1986) had a sensitivity of 50 epg, introducing substantial variability in the FEC results as the identification of one egg might represent counts of between 1 and 99 epg. This cannot be accounted for in calculation of anthelmintic efficacy based on population mean values [35]. Use of more refined salt floatation egg counting methods, such as the cuvette technique with a potential sensitivity of 1 epg [36], to overcome this problem would have required equipment that was unavailable for use in the field scenario of our study. However, in terms of our aim of using FECs as a means of engagement with livestock health, the precision of the estimate of anthelmintic efficacy is less important than simply determining whether or not the drug worked. Our data did not allow trends in FECs with calf age, sex or location to be evaluated, which would have aided our understanding of the problem. Determining whether anthelmintic inefficacy was due to poor drug quality or anthelmintic resistance was beyond the scope of our study.

## Conclusions

In summary, faecal egg counting was a practical means of engaging with livestock producers in rural communities in southern Malawi. Our study highlights poor health of village and dairy co-operative calves, coupled with a lack of veterinary services, animal health education and surveillance regarding cattle health. We have shown unexpected high trichostrongyle nematode FECs and poor anthelmintic efficacy of albendazole, highlighting the immediate need to address sustainable medicines use for the management of gastrointestinal nematode parasites; and highlighting the need for diagnostic tools, disease surveillance, regulation of veterinary medicine supply and education.

## Abbreviations

PPP international $: Purchasing Power Parity (international $)
AVO: Assistant Veterinary Officer
CAHW: Community Animal Health Worker
DAHLD: Department of Animal Health and Livestock Development
EPA: Extension Planning Area
EPG: Eggs Per Gram
FEC: Faecal Egg Count
FECR: Faecal Egg Count Reduction PPP international
FLT: Farm Livestock Technician
SHMPA: Shire Hills Milk Producers Association
